# Delays in arrival and treatment in emergency departments: Women, children and non-trauma consultations the most at risk in humanitarian settings

**DOI:** 10.1371/journal.pone.0213362

**Published:** 2019-03-05

**Authors:** Isabel Beltrán Guzmán, Julita Gil Cuesta, Miguel Trelles, Omar Jaweed, Sophia Cherestal, Joris Adriaan Frank van Loenhout, Debarati Guha-Sapir

**Affiliations:** 1 Centre for Research on the Epidemiology of Disasters, Institute of Health and Society, Université Catholique de Louvain, Brussels, Belgium; 2 Operational Centre Geneva, Médecins Sans Frontières, Geneva, Switzerland; 3 Operational Centre Brussels, Médecins Sans Frontières, Brussels, Belgium; 4 Mission Afghanistan, Médecins Sans Frontières, Kunduz, Afghanistan; 5 Mission Haiti, Operational Centre Brussels, Médecins Sans Frontières, Port-au-Prince, Haiti; Duke-NUS Medical School, SINGAPORE

## Abstract

**Introduction:**

Delays in arrival and treatment at health facilities lead to negative health outcomes. Individual and external factors could be associated with these delays. This study aimed to assess common factors associated with arrival and treatment delays in the emergency departments (ED) of three hospitals in humanitarian settings.

**Methodology:**

This was a cross-sectional study based on routine data collected from three MSF-supported hospitals in Afghanistan, Haiti and Sierra Leone. We calculated the proportion of consultations with delay in arrival (>24 hours) and in treatment (based on target time according to triage categories). We used a multinomial logistic regression model (MLR) to analyse the association between age, sex, hospital and diagnosis (trauma and non-trauma) with these delays.

**Results:**

We included 95,025 consultations. Males represented 65.2%, Delay in arrival was present in 27.8% of cases and delay in treatment in 27.2%. The MLR showed higher risk of delay in arrival for females (OR 1.2, 95% CI 1.2–1.3), children <5 (OR 1.4, 95% CI 1.4–1.5), patients attending to Gondama (OR 30.0, 95% CI 25.6–35.3) and non-trauma cases (OR 4.7, 95% CI 4.4–4.8). A higher risk of delay in treatment was observed for females (OR 1.1, 95% CI 1.0–1.1), children <5 (OR 2.0, 95% CI 1.9–2.1), patients attending to Martissant (OR 14.6, 95% CI 13.9–15.4) and non-trauma cases (OR 1.6, 95% CI 1.5–1.7).

**Conclusions:**

Women, children <5 and non-trauma cases suffered most from delays. These delays could relate to educational and cultural barriers, and severity perception of the disease. Treatment delay could be due to insufficient resources with consequent overcrowding, and severity perception from medical staff for non-trauma patients. Extended community outreach, health promotion and support to community health workers could improve emergency care in humanitarian settings.

## Introduction

The purpose of an effective emergency medical system is to provide timely medical care to prevent death or disability [1]. Delays in arrival to the emergency department (ED), and in receiving treatment can lead to severe negative outcomes and poor prognosis [2].

The time of delay in medical care has been defined as the interval between the onset of symptoms and the moment of treatment [3]. The length of this delay includes pre and in hospital times and is determined by a number of factors [4]. Among those inherent to the population seeking care, demographic characteristics, socioeconomic status and ethnicity are the most frequently described [5,6]. However, less-studied factors such as the type of pathology could also play a role. Factors related to health systems include availability of services, accessibility of health structures, acceptability and sufficient hospital resources [7]. The interaction of both individual and external factors could cause longer delays in arrival and treatment.

Delay in medical care is particularly concerning in low-resource settings, such as low-income countries (LIC) and humanitarian settings (i.e. areas affected by natural disasters or conflicts). In such contexts, the health care system often lacks resources or has been disrupted. Additionally, some communities may have a higher vulnerability due to pre-existing health disparities [8]. Médecins Sans Frontières (MSF), also known as Doctors Without Borders in English, is an international, independent, medical organization that works in humanitarian and low-resource settings [9]. Populations in these settings are highly vulnerable, there is a high demand for medical care services and timely access to emergency care is more challenging.

Previous studies assessing delays in receiving medical care focused on factors related to delays in arrival to health facilities in LIC [4,10,11] or delays in treatment within EDs and related negative outcomes [12–16]. The present study aims to fill the gap in knowledge through analysing simultaneously factors associated with both, delay in arrival and in treatment within the same settings. The analysis of populations from the EDs of three different countries in humanitarian settings could reveal shared challenges and thus give a wider perspective. The aim of our study is to provide findings relevant for the development of integrated health strategies. These strategies promise to improve emergency medical care for vulnerable populations, when factor associated with common challenges in low-resource settings are tackled.

## Methodology

This was a cross-sectional study based on routine data collected from three hospitals during the period 2013–2015 (Gondama December 2013 –October 2014; Martissant January 2015 –December 2015 and Kunduz January 2014 –August 2015). The data were reported to Médecins Sans Frontières, Operational Centre Brussels (MSF-OCB).

### Study settings

*Kunduz Trauma Centre* was located in Kunduz in North Afghanistan. In response to the lack of trauma treatment capacity in the area, the centre opened in August 2011. The centre had an ED, three operating theatres and an intensive care unit (ICU). It was equipped with X-ray, laboratory facilities and pharmacy. Patients also benefited of mental health, physiotherapy and out-patient follow-up. The population attending the centre was from Kunduz and the neighbouring provinces (Takhar, Baghlan and Badakhshan). On October 3rd, 2015, Kunduz Trauma Centre was destroyed by an airstrike.

*Martissant 25 Emergency Centre* is located in Martissant, one district in Port-au-Prince, Haiti. The centre has been operational since December 2006, benefiting the residents of the western area of Port-au-Prince. It offers mainly stabilization and emergency referrals through its ED and has a small capacity (six beds) short-length stay ward [17], functioning mainly as referral centre. The centre is equipped with pharmacy and X-ray departments and has a referral system with fully equipped ambulances.

*Gondama Referral Centre* is located in Gondama village, Bo District, in Sierra Leone. It was built by MSF-OCB in April 2003. The target population consists primarily of children under 15 years from the Bo district. The centre has an ED, neonatology, inpatient and intensive care wards; and a referral system for complicated paediatric surgeries to a hospital in Freetown. In October 2014, MSF suspended activities in the centre in order to respond to the Ebola outbreak and instituting several Ebola centres across the country.

### Study population

We included all patients who attended the ED of these hospitals during the intervals of time between 2013–2015 (Gondama December 2013 –October 2014; Martissant January 2015 –December 2015 and Kunduz January 2014 –August 2015). The inclusion criterion was that records were complete for sex, age group, hospital, SATS category, type of diagnosis (trauma or non-trauma) and outcome.

### Data collection and management

The data were obtained from the consultations registered by the emergency departments of the three hospitals. This information was regularly reported to MSF—OCB who provided it for the study. No personal information from the patients was included. The data processing was performed using Excel 2011, version 14.2.6.

### Variable definitions

The time of delay can be divided in two phases: “delay in arrival” or pre-hospital delay [3], and “delay in treatment”. Delay in arrival to the ED was defined as the duration from onset of symptoms to triage. It was categorised as a “delay ≤ 24 hours” or “delay > 24” hours from onset of symptoms, until being triaged in the ED. This threshold was selected arbitrarily because there is no consensus on this interval, taking into consideration different pathologies. Delay in treatment refers to ED or in-hospital delay and is represented by the duration of waiting times before receiving treatment but after arriving in the ED and being triaged. Delay in treatment was recorded when the target time for treatment was exceeded according to the assigned triage urgency category based on the South African Triage Scale (SATS) [18] used in the three hospitals (Table 1).

**Table 1:** South African Triage Scale (SATS) urgency categories and target times to begin treatment.

COLOUR	RED	ORANGE	YELLOW	GREEN	BLUE
Target time to treat	Immediate	Less than 10 mins	Less than 60 mins	Less than 240 mins	

Adapted from the SATS training manual 2008.

Diagnoses were categorized into trauma (violent or accidental) and non-trauma based on the surveillance code given to pathologies in MSF projects. Outcome was categorized into three values: as “admitted” if a patient was hospitalised after being triaged and having received medical consultation in the ED; as “death” if the patient died after being triaged, but before being hospitalised; as “Others” for cases who were either discharged home, were referred to another facility, or left the ED before consultation.

### Data analysis

Statistical analyses were performed with RStudio, Version 0.99.902. We calculated the proportion of patients by sex, age group, SATS category and outcome for the three facilities combined, as well as for each hospital separately. We also calculated the proportion of cases with “delay on arrival” and “delay in treatment” by hospital, sex, age group, SATS, category, type of diagnosis (trauma or non-trauma) and outcome. Comparisons between groups for categorical variables were performed using Chi-square tests. We reported only significant results (p-value <0.05).

We used a multinomial logistic regression model (MLR) to analyse factors associated with delay in arrival and delay in treatment. From the different variables, we included only those which contributed significantly to the model. The Odds Ratios (ORs) were reported with 95% confidence intervals (CI).

### Ethics

Confidentiality of information was ensured during all processing of data entering and analysis. Data were collected during routine treatment and then anonymized; therefore, individual consent was not required. This research fulfilled the exemption criteria set by the Médecins Sans Frontières Ethics Review Board (ERB) for a posteriori analyses of routinely collected clinical data and thus did not require MSF ERB review. It was conducted with permission from (Medical Director, Operational Centre) Médecins Sans Frontières. Following the national ERB regulations, Ethical approval was obtained from the Haiti ERB. (Ref. 1718–70)

## Results

### Characteristics of the consultations

We included 95,025 consultations (4,647 from Gondama, 40,482 from Kunduz and 49,896 from Martissant). Males represented 65.2% of the total sample. Table 2 summarises the characteristics by hospital and for the total sample. In Kunduz the main related diagnoses were as expected trauma accidental (87.9%) and violence (12.1%). Martissant was also mainly attended by trauma cases (58.7% and 9.9% accidental and violence, respectively). Gondama had as main complain severe malaria (38.8%), followed by uncomplicated malaria (17.3%). The overall mortality was 0.5% for the three hospitals combined.

**Table 2:** Baseline characteristics of the consultations by hospital during the period 2014–2015.

Hospital	Gondama		Kunduz		Martissant		Total	
	n	%	n	%	n	%	n	%
**Total**	4,647	4.9	40,482	42.6	49,896	52.5	95,025	100.0
**Sex**								
Female	2,072	44.6	9,457	23.4	21,580	43.6	33, 109	34.8
Male	2,575	55.4	31,025	76.6	28,316	56.8	61, 916	65.2
Age Group*								
0 to 4	4,095	88.1	3,668	9.1	7,276	14.6	15,039	15.8
5 to 17	551	11.9	13,885	34.3	30,912	20.4	24,624	25.9
18 to 65	1	0.0	21,808	53.9	30,912	62.0	52,721	55.5
Above 65	-	-	1,121	2.8	1,520	3.0	2,641	2.8
SATS Category**								
Red (Immediate)	1,601	34.5	1,741	4.4	1,568	3.1	4,910	5.2
Orange (<10)	1,665	35.8	9,912	24.5	10,083	20.2	21,660	22.8
Yellow (<60)	999	21.5	20,060	49.6	23,923	48.0	44,982	47.3
Green (<240)	357	7.7	8,534	21.1	14,293	28.7	23,184	24.4
Blue	25	0.5	235	0.1	29	0.1	289	0.3
**Outcome**								
Admitted	3,737	80.4	4,901	12.1	733	1.5	9,371	9.9
Died	87	1.9	275	1.4	86	0.1	448	0.5
Others**†**	823	17.7	35,313	87.2	49,084	98.4	85,220	89.6

*Age reported in years.

** SATS Category: South African Triage Scale target time for treatment in minutes according to urgency code

**†** Others: cases who were referred to another facility, left the ED before consultation or were discharged home from the ED.

### Delay in arrival

Delay in arrival was present in 27.8% of the total cases. Characteristics associated with delay in arrival by hospital, sex, age, triage category, diagnosis and outcome are presented in **Table 3**. By hospital, Gondama showed the highest proportion of delay (96.4%). Females had higher proportion of delay in in Kunduz (31.6%.) and Martissant (26.2%). By age group children under five accounted for 87.5% of the total population with delay in arrival in Gondama. In Kunduz, this proportion was for the group above 65 years (36.6%), while in Martissant it was for children under five (40.0%). Red and orange triage categories accounted for 69.3% of the total delays in arrival in Gondama. In Kunduz the highest proportion of delays was for green cases (41.5%) and in Martissant, for red and orange cases (42.1% and 26.1%, respectively). Regarding diagnosis, Martissant showed higher proportion of delays for non-trauma cases (43.6%). By outcome, admitted had the highest proportion of delay in Kunduz (16.7%) and Martissant (64.9%).

**Table 3:** Delays in Arrival by hospital, sex, age, SATS category, diagnosis and outcome during the period 2013–2015.

	Gondama			Kunduz			Martissant		
	≤ 24hrs		>24hrs		Total	≤ 24hrs		>24hrs		Total	≤ 24h		>24hrs		Total
	n	%	n	%	N	n	%	n	%	N	n	%	n	%	N
**Total**	167	3.6	4,480	96.4	4,647	30,175	74.5	10,304	25.5	40,479	38,257	76.7	11,639	23.3	49,896
**Sex**	** **	** **													
Female	74	3.6	1,998	96.4	2,072	6,473	68.4	2,984	31.6	9,457	15,929	73.8	5,651	26.2	21,580
Male	93	3.6	2,482	96.4	2,575	23,702	76.4	7,320	23.6	31,022	22,328	78.9	5,988	21.1	28,316
Age Group*	** **	** **				** **	** **				** **	** **			
0 to 4	167	4.1	3,928	95.9	4,095	2,899	79.1	768	20.9	3,667	4,369	60.0	2,907	40.0	7,276
5 to 17	0	-	551	100.0	551	10,762	77.5	3,122	22.5	13,884	7,939	77.9	2,249	22.1	10,188
18 to 65	0	-	1	100.0	1	15,803	72.5	6,004	27.5	21,807	24,786	80.2	6,126	19.8	30,912
Above 65	-	-	-	-	-	711	63.4	410	36.6	1,121	1,163	76.5	357	23.5	1,520
SATS Category**	** **	** **				** **	** **				** **	** **			
Red (Immediate)	119	7.4	1,482	92.6	1,601	1,655	95.1	86	4.9	1,741	908	57.9	660	42.1	1,568
Orange (<10)	39	2.3	1,626	97.7	1,665	8,610	86.9	1,301	12.1	9,911	7,449	73.9	2,634	26.1	10,083
Yellow (<60)	7	0.7	992	99.9	999	14,768	73.6	5,290	26.4	20,058	19,004	79.4	4,919	20.6	23,923
Green (<240)	2	0.6	355	99.4	357	4,909	57.5	3,625	41.5	8,534	10,869	76.0	3,424	24.0	14,293
Blue	0	0.0	25	100.0	25	233	99.1	2	0.9	235	27	93.1	2	6.9	29
**Diagnosis**	** **	** **				** **	** **				** **	** **			
Trauma	1	1.5	67	98.5	68	30,171	74.5	10,302	25.5	40,473	29,003	86.6	4,496	13.4	33,499
Non-trauma	166	3.6	4,413	96.4	4,579	4	66.7	2	33.3	6	9,254	56.4	7,143	43.6	16,397
**Outcome**	** **	** **				** **	** **				** **	** **			
Admitted	164	4.4	3,573	95.6	3,737	4,084	83.3	817	16.7	4,901	257	35.1	476	64.9	733
Died	0	0.0	87	100.0	87	271	98.5	4	1.5	275	77	89.5	9	10.5	86
Others†	3	0.4	820	99.6	823	25,820	73.1	9,483	26.8	35,303	37,923	77.2	11,154	22.7	49,077

* Age reported in years.

** SATS Category: South African Triage Scale target time for treatment in minutes according to urgency code

† Others: cases who were referred to another facility, left the ED before consultation or were discharged home from the ED.

### Delay in treatment

Delay in treatment was present in 27.2% of the total number of patients. Females had higher proportion of delay in treatment in Gondama and Martissant (38.6% and 50.1%, respectively). By age group, a higher proportion was observed for children under five in Gondama and Martissant (39.0% and 65.2%, respectively), while in Kunduz was for population above 65 years (5.8%). Concerning triage category, red cases showed higher proportion of delay in treatment for the three facilities (Gondama 87.6%, Kunduz 70.8% and Martissant 85.2%). Non-trauma cases had higher delay in all three facilities (Gondama 37.1%, Kunduz 16.7% and Martissant 57.1%). By outcome, admitted cases also showed higher proportion of delays (Gondama 41.2%, Kunduz 22.6% and Martissant 85.0%). Factors associated with delay in treatment by hospital, sex, age, triage category, diagnosis and outcomes are shown in Table 4.

**Table 4:** Delay in Treatment by hospital, sex, age, SATS category, diagnosis and outcome during the period 2013–2015.

	Gondama					Kunduz					Martissant				
	No Delay		Delay		Total	No Delay		Delay		Total	No Delay		Delay		Total
	n	%	n	%	N	n	%	n	%	N	n	%	n	%	N
**Total**	2,870	63.0	1,689	37.0	4,559	37,503	94.9	2,018	5.1	39521	21,164	52.4	19,251	47.6	40,415
**Sex**	** **	** **													
Female	1,249	61.4	784	38.6	2,033	8,928	96.5	328	3.5	9,256	8,659	49.9	8,681	50.1	17,340
Male	1,621	64.2	905	35.8	2,526	28,575	94.4	1,690	5.6	30,265	12,505	52.8	10,570	45.8	23,075
Age Group*	** **	** **				** **	** **				** **				
0 to 4	2,449	61.0	1,568	39.0	4,017	3,462	96.3	133	3.7	3,595	2,119	34.8	3,973	65.2	6,092
5 to 17	420	77.6	121	22.4	541	12,941	95.5	611	4.5	13,552	4,248	49.9	4,270	50.1	8,518
18 to 65	1	100.0	0	-	1	20,070	94.3	1,211	5.7	21,181	14,193	57.6	10,438	42.4	24,631
Above 65	0	-	0	-	0	1,030	94.2	63	5.8	1,093	604	51.4	570	48.6	1,174
SATS Category**	** **	** **				** **	** **				** **				
Red (Immediate)	194	12.4	1,375	87.6	1,569	489	29.2	1,186	70.8	1,675	180	14.8	1,040	85.2	1,220
Orange (<10)	1,368	83.2	277	16.8	1,645	9,039	93.6	620	6.4	9,659	1,544	20.2	6,095	79.8	7,639
Yellow (<60)	936	96.6	33	3.4	969	19,442	99.0	192	1.0	19,634	8,103	41.9	11,252	58.1	19,355
Green (<240)	348	98.9	4	1.1	352	8,305	99.8	20	0.2	8,325	11,314	92.9	864	7.1	12,178
Blue	24	100.0	0	-	24	228	100.0	0	-	228	23	100.0	0	-	23
**Diagnosis**	** **	** **				** **	** **				** **				
Trauma	46	68.7	21	31.3	67	37,498	94.9	2,017	5.1	39,515	15,876	56.5	12,221	43.5	28,097
Non-trauma	2,824	62.9	1,668	37.1	4,492	5	83.3	1	16.7	6	5,288	42.9	7,030	57.1	12,318
**Outcome**	** **	** **				** **	** **				** **				
Admitted	2,161	58.8	1,516	41.2	3,642	3,656	77.4	1,069	22.6	4,725	88	15.0	497	85.0	585
Died	42	66.7	42	33.3	84	241	89.9	27	10.1	268	43	64.2	24	35.9	67
Others†	667	83.6	131	16.4	798	33,606	85.0	922	2.33	34,528	21,033	53.8	18,730	48.0	39,043

* Age reported in years.

** SATS Category: South African Triage Scale target time for treatment in minutes according to urgency code

† Others: cases who either referred to another facility, left the ED before consultation or were discharged home from the ED.

### Characteristics associated with delay in arrival and treatment

For the MLR analyses we included sex, age, hospital and diagnosis. SATS category and outcome did not contribute significantly, and were not included in the models. The model for delay in arrival showed that females had a higher risk of delay compared to males (OR 1.2, 95% CI 1.2–1.3). For the different age groups, children less than five showed the highest risk of delay (OR 1.4, 95% CI 1.4–1.5) compared to adults. By hospital, patients attending to Gondama showed the highest risk (OR 30.0, 95% CI 25.6–35.3) of having delay in arrival compared to patients attending to Martissant. Patients with non-traumatic diagnosis had over 4 times a higher risk (OR 4.6, 95% CI 4.4–4.8) of delay compared to those with trauma. Table 5 shows the OR for all the variables included in the model.

**Table 5:** Characteristics associated with delay in arrival and treatment in the selected hospitals during the period 2013–2015.

Independent Variable	Arrival Delay †*		Treatment Delay‡ **	
	OR	95% CI	OR	95% CI
**Sex**				
Male	Ref	Ref	Ref	Ref
Female	1.2	1.2–1.3	1.1	1.0–1.1
Age^**§**^				
18 to 65	Ref	Ref	Ref	Ref
0 to 4	1.4	1.4–1.5	2.0	1.9–2.1
5 to 17	1.0	1.0–1.1	1.3	1.2–1.3
Above 65	1.3	1.1–1.4	1.2	1.1–1.3
**Hospital**				
Martissant	Ref	Ref	14.6	13.2–15.4
Kunduz	2.3	2.2–2.4	Ref	Ref
Gondama	30.0	25.6–35.3	0.3	0.3–0.3
**Diagnosis**				
Trauma	Ref	Ref	Ref	Ref
Non—Trauma	4.6	4.4–4.8	1.6	1.5–1.7

Odds Ratios from the Multinomial Logistic Regression models.

†Compared to ≤ 24 hours.

‡Compared to no Delay.

*McFadden R^2: 0.14 for the arrival delay model.

**McFadden R^2: 0.22 for the treatment delay model.

§ Age reported in years.

For delay in treatment, the model also showed a higher risk delay for females (OR 1.1, 95% CI 1.0–1.1) compared to males (Table 5). Also in this model, children less than five had the highest risk (OR 2.0, 95% CI 1.9–2.1) compared to adults. Patients in Martissant had a higher risk of delay in treatment (OR 14.6, 95% CI 13.9–15.4) compared to Kunduz. Non-trauma cases had a higher risk of receiving delayed treatment compared to trauma cases (OR 1.6, 95% CI 1.5–1.7).

## Discussion

Our study found significant associations between the factors assessed and delays, both in arrival and in receiving treatment. Females and children less than five had an increased risk of delay in arrival and treatment. For the hospitals, a greater proportion of patients attending Gondama had a delay in arrival, while patients seeking care in Martissant were most likely to receive delayed treatment. Non-trauma cases were found to have more delay in treatment compared to trauma cases. We present the discussion of each of the delays in two separate sections.

### Arrival delay

Access to health care is the result of factors related to the population (demand-side-factors factors) and the health system (supply-side-factors). In humanitarian settings, the complex interaction of these factors can result in access barriers for timely access to emergency care for vulnerable populations. We therefore describe the different factors identified affecting the accessibility in the three humanitarian settings included in our study.

Sex disparities when seeking medical care have been reported in different studies, showing that women are more likely to have longer delay in arrival compared to men [19]. The observed delays in Kunduz and Martissant reflect such disparities. This could be related to factors commonly obstructing access and acceptability for women. Among those, educational gaps, cultural behaviours and low socioeconomic status could play a major role. Illiteracy can cause of poor understanding of medical needs, symptoms and severity of diseases, as well as treatment options [20]. Low educational level is related to lower utilization of institutional health services, negatively affecting women’s health (e.g. maternal mortality due to complications of home births) [21]. This is relevant considering that in LIC, girls are more likely to be excluded from education than boys [22]. Cultural behaviours related domestic responsibilities and beliefs surrounding female conditions (e.g. pregnancy and delivery) also relate to delays in arrival for women [23,24]. Finally, in contexts without appropriate free health coverage, low socioeconomic status, lack of free medical care and of health centres within walking distance contribute to delays in seeking medical attention for women [25,26].

In low-resource settings where the overall educational level is low, health education and promotion are essential strategies to increase awareness and reduce health risks [27,28]. These strategies should target women, mainly illiterate ones focusing on prevention, emergency symptoms, availability of health services, and the importance of timely attendance to them. Health strategies should also integrate community traditions and beliefs. Collaboration with traditional birth attendants (TBAs) has shown to increase female health education, improvement in skilled birth attendance and timely access to obstetric emergency care due to early recognition of complications [29].

Maternal access barriers to health and cultural behaviours could determine arrival delay for children [30]. Children are especially vulnerable as they depend entirely on their caregiver, most often the mother, and their health is closely related to hers [31]. In consequence, delays in arrival to the ED for children are linked to barriers faced by women. Maternal schooling and infant mortality are also closely related [32]. This explains our findings in Martissant where both females and children under-five showed arrival delay. Convenience and availability of non-institutional options such as use of household near herbs, reduced remedies costs and cultural linkages also relate to delays [33]. Such preferences could also explain the observed delays in our study for children less than five in Martissant. The National Health Policy, elaborated by the Haitian government in 2012 [34], is a 25-year plan to reduce morbidity and mortality. Among its objectives, contemplates the articulation of modern and traditional medicine and promise to be an integral strategy.

The Free Health Care Initiative (FHCI), introduced in 2010 by the Sierra Leone Government, removed user fees for pregnant and lactating women, and children less than five years old [35]. However, in some areas, traditional medicine is still preferred when a child is ill, due to lack of access to government services, indirect costs and unavailability of drugs in the facilities [36]. When non-institutional alternatives are ineffective, the decision to attend a health institution is taken. This process inevitably extends the delay in arrival to the ED, as shown in our study for Gondama.

As a result of the aforementioned factors, timely access to health care for children could be improved through health education targeting illiterate women and improvement of females´ health care access. Understanding of cultural behaviours, and more importantly, integrating traditional healers, will also reduce delays in arrival in this type of settings. This has been demonstrated to be effective when the healers were sensitised, informed, and worked in collaboration with institutional medical staff [37]. In addition, improving quality of care and treatment availability is essential to build trust in the communities and improve institutional health centres utilization for strategies like the FHCI to be effective.

Additional factors such as lack of emergency medical transportation and insecurity could add barriers to access for specific populations. This could explain the observed delays in Kunduz. In highly violent and insecure contexts, vulnerable populations such as women and elderly could postpone ED visits due to insecurity. In such environments, attendance to the ED would be reserved for pathologies perceived as life-threatening reflected by the number of green cases with delay in arrival. The Basic Package of Health Services (BPHS) implemented in 2003 and the Essential Package of Hospital Services (EPHS) in 2005 aimed to improve the quality of hospital services for Afghans [38]. Nevertheless, such policies may be insufficient in complex humanitarian settings due to access barriers related to violence, insecurity and lack of affordable transportation from remote areas [39]. Ultimately, knowing that accessibility factors are very context specific, a revision of the accessibility frameworks in humanitarian settings would be very useful to contextualize the operational research recommendations.

Delays in medical care have a negative impact in patients’ severity and outcomes (i.e. myocardial infarction, postpartum haemorrhage) [2,40]. This could explain the observed association in our study between red cases and admissions with delay in arrival. Many high-income countries have reduced prehospital times through improving ambulance response, improving paramedical skills to perform on site procedures, and even accounting for ambulance off-stretcher intervals [41,42]. In low-resource settings, it might not be feasible to implement these strategies and patients rely entirely on their own means to attend to an ED.

In resource constricted settings, the implementation of mobile clinics is a feasible strategy to increase coverage of remote populations with periodical consultations and health education, identification of potential cases and referring patients in case of emergency. Community health workers (CHWs) could also respond to many of these needs. Health education programs using CHWs have proved to positively impact health of the population, principally when they involve the community participation and use focus groups [43,44]. In remote areas with no transportation available or locations where security may not allow population movements, particularly for women, lay health workers could be the most appropriate alternative. They understand the cultural background, and may be better perceived and accepted by the community [45]. With adequate training and minimal equipment, they could be able to serve as first responders and link with the nearest hospital in case of emergency care needed.

The observed arrival delay in Martissant for non-traumatic cases could also be associated to the perceived severity. A patient with a traumatic injury could seek medical care earlier out of a concern of severe injury. However, no studies were found to support this assumption. Qualitative studies assessing the perception of the severity of a disease related to trauma or not among different populations are needed to gain a better understanding of the underlying cause.

### Treatment delay

Treatment delay could be related to lack of resources and insufficient health facilities availability, resulting in overcrowding. The implementation of triage scales like the SATS in low resource settings, have shown to maximize use of time and resources when sorting out and prioritizing patients [17,46]. Nevertheless, once a patient has been categorized, the time to finally receiving treatment will depend on patients’ flow through the ED. This flow is the result of the balance between input-throughput-output, a disturbance at any of these stages due to insufficient resources will result in overcrowding [47].

Overcrowding is associated with negative outcomes, patient frustration, medical staff burnout and errors [12]. It has become one of the main issues in EDs worldwide, mainly in low-resource settings, where the lack of available medical facilities concentrates numerous patients in a limited number of hospitals [48]. Additionally, if the number of medical staff or beds are insufficient, patient flow would be affected, increasing waiting times, delaying treatment and increasing the risk of negative outcomes. In Martissant, where we observed the higher proportion of delay in treatment, this may be the result of overcrowded EDs due to a high demand, lack of sufficient staff and available beds to provide emergency care, or a combination of these. Outreach strategies including mobile clinics could reduce overcrowding in the functional hospitals and reduce risk of negative outcomes [49–51].

In low-resource hospitals, an increase in the demand for urgent treatment due to numerous wounded patients, or seasonal peaks of infectious diseases (e.g. malaria, pneumonia) could exceed the ED immediate treatment capacity. These urgent cases have minimal target time for treatment and managing more than a few simultaneously may not be achievable in such settings. This could explain the observed high number of red cases with delay in treatment. Lay CHWs could be part of health improvement strategies: if well trained, they can provide initial basic management for medical conditions with rapid progression (e.g. mild to severe malaria), helping to reduce the pace of the clinical evolution to severe conditions as well as to reduce the need for hospitalizations.

No literature was found to explain why females and children less than five have a higher risk of delayed treatment. Future studies in similar contexts should be performed to understand such phenomena, which is critical due to the high vulnerability of these groups.

Concerning the higher delay in treatment we observed for non-trauma diagnoses, greater delay to receive medical care could be explained by the perceived severity from the medical staff. Trauma patients have a known high mortality [52] and might be prioritized over other patients. Further qualitative studies assessing the decision-making process and urgency perception by medical staff between trauma and non-trauma cases could clarify this.

It is relevant to underline that despite the observed delay to receive treatment, the overall ED mortality was very low compared to other studies evaluating overall ED mortality rates in low resource settings [53]. We could imply that the immediate impact of such a delay was not important enough to cause death. Nevertheless, this study did not account for non-immediate outcomes, length of hospitalization and associated costs or disability after being discharged.

## Strengths

The study was based on a large sample from three hospitals (N = 95,025). All three of them use the same MSF standard treatment protocols and the same triage system (SATS). This makes the triage categorization and patient management standard, and therefore comparable, across the three settings. Additionally, even though the hospitals are from countries in utterly different geographical regions (South-Central Asia, West Africa and the Caribbean), they all share low-resource setting characteristics. Subsequently, the results from this study provide information relevant to other countries with similar contexts.

## Limitations

This was a retrospective study based on routinely collected programme data, therefore, no additional factors were available to be included in the analysis that could have possibly be associated with delays. Staff to patient ratio, also with respect to different shifts (day/night, weekends, etc.), could result in a different capacity to promptly treat patients. Bed occupancy was not known, while integrating it to the analysis would allow to evaluate its relationship with delays in these hospitals. Social interactions and patient expectations could have an influence in the medical decision to admit patients, but were difficult to assess in a study of this nature. Additionally, no information concerning availability and capacity of health facilities in the area was considered.

There is no consensus on the ideal minimum arrival delay for all non-trauma cases and trauma-related injuries, we therefore dichotomised this interval to draw a cut-off for our analysis, but we will not be able to compare it. We did not included severity in trauma cases (represented by triage category) in our model because our outcome measure was also defined by triage code and we considered its inclusion conflicting. Finally, no follow-up information was available, to know hospitalization outcomes that could be potentially caused by arrival and treatment delays.

## Conclusions

The findings in the three hospitals in our study document persistent delays in arrival and treatment in EDs in humanitarian settings. These findings resemble other studies and provides further evidence on unequal care for women and children. We hypothesize this could be due to the complex interaction on accessibility of individual factors (education, socioeconomic status, cultural behaviours and perception of the disease severity) and health system factors (availability and access to health services, and lack of sufficient hospital resources causing overcrowding).

The development of health strategies should include health education targeting woman, particularly illiterate ones, to improve female and infant health. They should also include information concerning severe symptoms and the importance of promptly seeking medical care.

The observed delays in treatment in Martissant hospital could relate to overcrowding due to exceeded ED capacity. Outreach interventions and community health workers can improve community coverage and reduce overcrowding.

No literature was found to explain the observed associations between the demographic characteristics (i.e. being female or infant) and non-trauma pathology with delay in treatment. Further studies assessing such delays are necessary to improve quality emergency care for these groups.
